# Acupuncture and related therapies for poststroke insomnia: A protocol for systematic review and network meta-analysis

**DOI:** 10.1097/MD.0000000000025039

**Published:** 2021-03-05

**Authors:** Chao Zhan, Zhao-Duan Hu, Yan Zhao, Xiao-Ming Fang, Wei Cheng, Song Lu, Zhi-Wei Chen

**Affiliations:** a Hubei University of Chinese Medicine, Wuhan; b Huangshi Traditional Chinese Medicine Hospital, Huangshi, Hubei, China.

**Keywords:** acupuncture, insomnia severity index (ISI), network meta-analysis (NMA), Pittsburgh sleep quality index (PSQI), poststroke insomnia, self-rating anxiety scale (SAS), self-rating depression scale (SDS)

## Abstract

**Objective:**

To compare and evaluate the clinical effects on patients with poststroke insomnia of various acupuncture and acupuncture-related therapies.

**Methods:**

In order to analyze the direct and indirect evidence from related studies, we used network meta-analysis (NMA). In order to collect randomized controlled trials (RCTs) of acupuncture and related therapies in the treatment of poststroke insomnia, 3 English and 4 Chinese databases were searched. After 2 researchers independently screened the literature, extracted the information, and assessed the probability of bias in the included studies, the data was analyzed using Stata15.0 and WinBUGS1.4.3 software.

**Results:**

Based on the existing data, the pros and cons of different acupuncture-related therapies are compared extensively, the effectiveness of different acupuncture-related therapies is ranked compared to drugs with hypnotic effect in poststroke insomnia care, and the best methods or combinations of acupuncture intervention are summarized.

**Conclusion:**

This study will provide new evidence for the safety and effectiveness of acupuncture-related therapies in the treatment of poststroke insomnia, and may be helpful for clinicians, poststroke insomnia patients, and clinical guideline makers to choose the optimal combination of acupuncture for the treatment of poststroke insomnia.

**Registration Number:**

INPLASY202120028.

## 1 Introduction

Insomnia is mainly manifested by shorter sleep duration and weakened sleep depth. Research reports show that the current incidence of insomnia in the world is about 30% to 35%, and about 1/3 of the adult population has insomnia symptoms, such as the longer time to fall asleep, ease to wake up after sleep, and difficulty falling asleep again after waking up.^[1]^ Poststroke insomnia is a common complication that is more likely to occur in stroke patients after the acute phase. In the process of neurological rehabilitation of stroke patients, about 56.5% of patients were accompanied by insomnia during the treatment.^[2]^ The long-term insomnia of stroke patients will not only aggravate the psychological burden, affect the therapeutic effect, but also may further develop into anxiety, depression, heart disease, hypertension, and so on,^[3]^ which may induce stroke again.^[4]^ Therefore, positive symptomatic treatment to improve the sleep quality of stroke patients is particularly important for the rehabilitation of stroke patients. At present, clinical treatment methods can be divided into drug therapy and nondrug therapy, among which the drugs are mainly benzodiazepine receptor agonist, melatonin receptor agonist, orexin receptor antagonist, and the west medicine with hypnotic effect.^[5]^ Nondrug therapy mainly includes external treatment of traditional Chinese medicine (TCM), physical therapy, and psychological therapy (such as massage, psychological counseling).^[6]^ Although the commonly used drugs in clinical practice can improve the symptoms of patients, long-term use of them may have many adverse reactions (such as drowsiness, drug resistance, dependence, etc.), so it is not recommended to use them for a long time. Acupuncture is a green therapy with high safety,^[7]^ studies shown that non-drug therapies such as acupuncture, moxibustion, auricular acupoint stimulation, and transcranial magnetic stimulation have good effects on patients with insomnia.^[8]^ With the increasing incidence of stroke, the number of patients suffering from insomnia after stroke is also increasing.^[9]^ In view of the shortcomings of drug treatments, there are more and more reports of poststroke insomnia patients using non-drug treatments with acupuncture as the main adjunct therapy, confirming the role of acupuncture in stroke and insomnia.

However, there is still a lack of clear comparative research between various acupuncture-related therapies given the large variety of acupuncture and the different emphasis on efficacy. The network meta-analysis (NMA) approach was used in this research to assess the effects of different acupuncture-related therapies on poststroke insomnia patients, which were intended to provide evidence-based medicine evidence to select the best combination of options.

## 2 Methods

It will be reported following the Preferred Reporting Items For Systematic Reviews And Meta-Analyses for Network Meta-Analysis Checklist (PRISMA-NMA) ^[10]^ and the Reporting Items for Systematic Reviews and Meta-Analysis of Acupuncture.^[11]^ This study has been registered with INPLASY, and registration number was INPLASY202120028.

### 2.1 Search strategies

Our search for literature was carried out from the establishment of the database until February 1, 2021, including 3 English databases: PubMed, EMBASE, Cochrane Library, and 4 Chinese databases: China Biology Medicine (CBM), China National Knowledge Infrastructure (CNKI), Wanfang Data, and Chinese Science Journal Database (VIP). A mixture of medical subject headings (MeSH) terms and free words were used to perform the search. Furthermore, to obtain more comprehensive resource, the references used in the medical literature have been supplemented retrospectively.

### 2.2 Inclusion Criteria

The published randomized controlled trials (RCT) of acupuncture-related therapies for the treatment of poststroke insomnia, irrespective of age and sex. Patients were expected to have passed the acute stage and to be diagnosed with poststroke insomnia or poststroke sleep disorders with clear diagnostic criteria. Different forms of acupuncture-related treatments, including acupuncture, electroacupuncture, warm acupuncture, atrial acupuncture stimulation, acupuncture injection, acupuncture catgut embedding, or a mixture of acupuncture and medications, have been included in the treatment group; the control group consists of different Western sleep aids or comparison between various acupuncture-related therapies. At least 1 of the following outcome measures should be included in the study results: Effectiveness Rate, Pittsburgh sleep quality index (PSQI), Insomnia Severity Index (ISI), Self-Rating Anxiety Scale (SAS), and Self-Rating Depression Scale (SDS). The language of the publication is limited to Chinese or English.

### 2.3 Exclusion criteria

1. Research on self-controlled or other non-RCTs;
2. research with unclear diagnostic criteria;
3. pre-clinical studies, case reports, systematic reviews and meta-analysis;
4. the report did not have clear original data, and contacting the author was unsuccessful;
5. there was no acupuncture-related therapy or other forms of acupuncture (such as transcutaneous electrical nerve stimulation or transcranial magnetic stimulation);
6. repeated research or research report results are the same.

### 2.4 Literature screening and data extraction

Two researchers independently conducted the literature screen, data extraction, and cross-check. In any case of disagreement, the third researcher would make the final determination. A unified data extraction table was used for data extraction, which included general information of the included literature:

1. the name of the first author, the journal name, the publication year, and so on;
2. baseline data of the subjects included in the literature: grouping, the sample size of each group, and subject's age;
3. intervention methods: type of intervention, treatment frequency, and treatment period;
4. risk bias-related factors: random method, allocation hiding, blinding;
5. outcome indicators before and after treatment data.

### 2.5 Risk assessment of bias in inclusion studies

Two researchers evaluated the included studies in accordance with the bias risk assessment tool recommended in the Cochrane Handbook 5.1.^[12]^

### 2.6 Statistical Analysis

Statistical analysis was performed using Stata 15.0 and WinBUGS 1.4.3 software.^[13–15]^ The effectiveness rate was categorically variable and the risk ratio was used to estimate its effect size. However, the PSQI, ISI, SAS, SDS scores were numerical variables, so the differences of the before and after treatment were used as effect size. In some trials, the change between baseline and after treatment failed to show, and the missing data was estimated using the formula recommended by the Cochrane Handbook 5.1.^[16]^ The Formula is as follows:

(1) $${\bar{X}}_{change}={\bar{X}}_{after\;treatment}-{\bar{X}}_{before\;treatment}$$

(2) $$SDchange=\sqrt{{\left(SD_{before-treatment}\right)}^{2}+{\left(SD_{after-treatment}\right)}^{2}-r\times SD_{before-treatment}\times SD_{after-treatment}}$$

First, Stata 15.0 was used to draw an NMA evidence relationship plot which is split and reconstituted into all paired two-arm tests if the literature is three-arm or more.^[17]^

Secondly, WinBUGS 1.4.3 was run to set the number of iterations for NMA to 50 000; to determine the accuracy of the closed-loop, 95% confidence interval (95% CI) of inconsistency factors (IF) was used. If there is 0 in the IF with 95% CI, it means that the direct and indirect proof is consistent; otherwise, it means that there is a greater risk of inconsistency.^[18]^

Thirdly, the Stata 15.0 software was used to build funnel plots to assess if the included studies had evidence of small sample effects.^[19]^

Finally, Stata 15.0 was used to produce the surface under the cumulative ranking curve (SUCRA) to represent the SUCRA scores for all treatments,^[20]^ with higher SUCRA scores indicating a higher treatment class.^[21]^

## 3 Discussion

Stroke is the leading cause of death and disability among adults in China, which is characterized by high morbidity, disability, mortality, and recurrence rate. The 2016 Global Burden of Disease (GBD) data shows that stroke is the first cause of years of life lost (YLL) in China.^[22,23]^ The incidence of stroke continues to rise, and shows a trend of getting younger. National stroke screening data showed that the incidence of the first-time stroke among people aged 40 to 74 increased from 189/100,000 in 2002 to 379/100,000 in 2013, with an average annual increase of 8.3%.^[24]^ The proportion of patients under the age of 70 in the stroke population continued to grow in China from 2005 to 2016.^[25]^ There are many factors that affect stroke, among which high blood pressure, diabetes, high blood fat, and unhealthy diet are high-risk factors for stroke.^[26]^ Stroke patients are often accompanied by a variety of dysfunction and inconvenience after passing the acute phase, of which 50% to 80% will suffer from insomnia.^[27]^ Hemiplegia, aphasia, sensory disorders, and other discomfort caused by stroke can easily induce insomnia, and the main clinical manifestations are difficulty in falling asleep and sleep interruption. Long-term insomnia can induce anxiety and depression, which will directly affect the recovery of stroke patients, and there is a possibility of further stroke.^[4]^ Therefore, timely and effective treatment for patients with insomnia after stroke is particularly important for the recovery of stroke.

Acupuncture, as a characteristic therapy of traditional Chinese medicine, has a good clinical effect on a variety of diseases. Considering the safety of acupuncture and the characteristics of no side effects, it can be used as a safe and effective supplementary alternative therapy for insomnia after stroke. Modern research and clinical efficacy also confirmed the effect of acupuncture on stroke and insomnia.^[28]^ The mechanism may be through acupuncture intervention, which could adjust the function of the brain cortex and brain stem ascending activating system,^[29]^ adjustment of the central nervous hormones and neurotransmitters,^[30]^ regulating endocrine and correcting nerve dysfunction,^[31]^ then achieve the purpose of keeping the balance between Yin and Yang and improving the quality of sleep. In addition, good doctor-patient interaction in the process of acupuncture is of great benefit to restore patients’ confidence in treatment, improve symptoms, relieve tension, and establish a doctor-patient relationship of mutual trust, ultimately improve the sleep quality of patients with insomnia after stroke.^[32]^

Based on the existing data, the pros and cons of different acupuncture-related therapies are compared extensively, the effectiveness of different acupuncture-related therapies is ranked compared to the hypnotic effect of Western medicine for poststroke insomnia, and the best methods or combinations of acupuncture intervention are summarized. As supportive alternative therapy for poststroke insomnia, this study may provide new evidence for the safety and efficacy of different acupuncture-related treatments.

## Author contributions

**Data curation:** Chao Zhan, Zhao-Duan Hu.

**Formal analysis:** Xiao-Ming Fang, Song Lu, Zhi-Wei Chen.

**Funding acquisition:** Yan Zhao, Chao Zhan.

**Methodology:** Chao Zhan, Zhao-Duan Hu.

**Resources:** Wei Cheng, Song Lu.

**Software:** Chao Zhan, Wei Cheng.

**Supervision:** Yan Zhao.

**Validation:** Xiao-Ming Fang.

**Writing – original draft:** Chao Zhan, Zhao-Duan Hu.

**Writing – review & editing:** Zhao-Duan Hu, Yan Zhao.
